# AI-Induced Vascular Ages Are a Measurable Residual Risk for Cardiovascular Diseases in the Japanese Population

**DOI:** 10.3390/jcm14134722

**Published:** 2025-07-03

**Authors:** Hikaru Ueno, Kotaro Uchida, Honoka Kawashima, Hiroto Hommo, Takuya Sugawara, Shintaro Minegishi, Lin Chen, Rie Sasaki-Nakashima, Tabito Kino, Kentaro Arakawa, Michiko Sugiyama, Koichi Tamura, Kiyoshi Hibi, Tomoaki Ishigami

**Affiliations:** 1 Department of Cardiology, Yokohama City University, Kanagawa 236-0004, Japankino-tabito@umin.ac.jp (T.K.);; 2 Department of Cardiology, Sir Run Run Hospital, Nanjing Medical University, Long Mian Avenue 109 Jiangning, Nanjing 210029, China; 3 Department of Cardio-renal Medicine, Yokohama City University, Kanagawa 236-0004, Japan

**Keywords:** vascular age, arterial stiffness, computer-aided vascular age, cardiovascular diseases

## Abstract

**Background:** Cardiovascular diseases (CVDs) remain a leading cause of morbidity and mortality, despite advances in treatment. Early detection of vascular aging is critical, as preclinical atherosclerosis often remains undiagnosed. AI-determined vascular age, originally developed using carotid-femoral pulse wave velocity (cf-PWV), may help to identify individuals at elevated risk. This study aimed to evaluate the clinical utility of an alternative AI-determined vascular age model based on the arterial velocity pulse index (AVI) and arterial pressure volume index (API) in a Japanese hospital-based cohort. **Methods:** This retrospective, exploratory study analyzed electronic health records of 408 patients from Yokohama City University Hospital. This study was approved by the Clinical Research Ethics Committee (approval numbers: B180300040, F240500007), and patient consent was obtained through an opt-out process. AI-determined vascular age was estimated using a Generalized Additive Model (GAM) with backward stepwise regression, substituting cf-PWV with AVI and API. Correlations with chronological age were assessed, and comparisons of cardiovascular and renal function markers were performed across age-stratified groups. **Results:** AI-determined vascular age showed a strong correlation with chronological age (*p* < 0.05). Significant differences were observed in cardiac diastolic function parameters, B-type natriuretic peptide (BNP), and estimated glomerular filtration rate (eGFR) between the highest and lowest quintiles of AI-determined vascular age. **Conclusions:** AI-determined vascular age using AVI and API appears to be a feasible surrogate for cf-PWV in clinical settings. This index may aid in stratifying vascular aging and identifying individuals who could benefit from early cardiovascular risk management.

## 1. Introduction

As Sir William Osler (1849–1919) famously stated, “A man is as old as his arteries”. Age is the most significant determinant of vascular aging and a critical risk factor for atherosclerotic cardiovascular complications. Unfortunately, aging is an irreversible process that worsens over time and has traditionally been regarded as an unmodifiable risk factor. However, if the opportunity arises to medicalize “age” as a residual risk factor, it may become possible to substantially suppress the incidence of atherosclerotic cardiovascular complications.

Healthy longevity, defined as successful aging without morbidity, is a critical healthcare priority in developed countries [1]. Advances in both therapeutic and diagnostic medical procedures, particularly in oncology, including molecularly targeted therapies such as immune checkpoint inhibitors, have significantly improved overall cancer mortality [2]. However, in contrast, cardiovascular diseases (CVDs) resulting from systemic atherosclerosis and arteriosclerosis in vital organs remain major life-threatening conditions despite the availability of advanced medical interventions and the involvement of highly trained healthcare professionals [3,4].

When severe, catastrophic cardiovascular (CV) events occur suddenly, substantial economic, social, and medical resources are required to rescue patients. This often leads to disparities in healthcare delivery between urban and rural areas. In clinical practice, catheter-based treatments for cardiovascular diseases are usually reserved for patients who present with symptoms, particularly those with acute coronary syndromes (ACS), whether in an acute or chronic stage. However, individuals with asymptomatic preclinical atherosclerosis and arteriosclerosis often go unnoticed and receive no significant medical intervention. Addressing this issue with effective solutions that are accessible worldwide is essential, as atherosclerosis and arteriosclerosis is a global health burden [5,6,7].

One of the major obstacles to achieving healthy longevity is cardiovascular complications [1,3]. Apart from arrhythmogenic diseases such as atrial fibrillation, which can lead to embolic events, most cardiovascular complications are considered atherosclerotic in nature. In Japan’s social security system, cardiovascular complications, along with musculoskeletal diseases and dementia, constitute a significant source of long-term care needs. Given the declining birthrate and aging population, these complications impose a severe financial burden on the national healthcare system.

Various procedures for detecting preclinical atherosclerosis and arteriosclerosis are already available in clinical settings, utilizing technologies such as ultrasound, pulse wave measurements, computed tomography (CT), magnetic resonance imaging (MRI), and a range of biochemical biomarkers. However, despite their widespread use, none of these procedures have been fully integrated into clinical practice with specific medical interventions, as stated above. Furthermore, chronological aging, which remains its strongest risk factor and contributor, is inevitable and cannot be medically controlled. Since atherosclerosis and arteriosclerosis are age-related conditions, this issue is a major obstacle for the clinical aspect of sub-clinical atherosclerosis and arteriosclerosis.

Vascular age has recently gained recognition as a meaningful parameter in cardiovascular risk stratification [5,8,9]. It reflects cumulative structural and functional changes within the arterial wall, most notably manifested as increased arterial stiffness [10]. This vascular stiffening contributes to elevated systolic pressure and excessive pulsatile load on end organs, such as the heart, brain, and kidneys. Among various available techniques, pulse wave velocity (PWV) is currently the most validated and widely accepted method for estimating vascular age, with strong predictive value for future cardiovascular events. Recently, Bruno et al. introduced AI-determined vascular age based on pulse wave velocity (PWV) technology, specifically cf-PWV, to classify individuals into three distinct categories as follows: early vascular aging (EVA), normal vascular aging (NVA), and supernormal vascular aging (SUPERNOVA) [11]. If individuals in the EVA (early vascular aging) group can be identified using AI vascular age and provided with appropriate medical care to transition to NVA (normal vascular aging), it may become possible to suppressively regulate ‘vascular aging,’ which is often considered an inevitable aging phenomenon, thereby extending healthy life expectancy [12].

Until now, we have conducted clinical research on the utility of new vascular indices using single-cuff oscillometric technology, PASESA (AVE-1500, Shisei Datum, Machida, Japan) [13,14,15,16]. Both the arterial velocity pulse index (AVI) and arterial pressure volume index (API) were found to be effective in detecting preclinical atherosclerosis and significantly predicting cardiovascular events in the Japanese population. In this study, we aimed to evaluate the performance and utility of AI-determined vascular age using these vascular indices, instead of cf-PWV, in a Japanese cohort.

## 2. Materials and Methods

The subjects of this study were patients who consecutively visited the Department of Cardiology at Yokohama City University Hospital, a public university corporation, with the first cohort consisting of those who visited between 10 January 2019 and 8 April 2019 (n = 110), and the second cohort consisting of those who visited between 13 May 2013 and 30 March 2015 (n = 298). Age, sex, height, weight, and BMI were recorded, along with systolic blood pressure, diastolic blood pressure, pulse rate, and AVI/API, measured three times on a single occasion using the AVE-1500 (Shisei Datum, Machida, Japan). The evaluation parameters included estimated glomerular filtration rate (eGFR), serum lipids (LDL-C, HDL-C), HbA1c, BNP, and comorbidities (diabetes mellitus, dyslipidemia, chronic kidney disease, and ischemic heart disease). The participants underwent echocardiography and renal ultrasonography during the data registration period, and assessments of cardiac systolic function, cardiac diastolic function, and renal arterial hemodynamic parameters were performed. This study was approved by the Clinical Research Ethics Committee of Yokohama City University, a public university corporation, under approval numbers “B180300040” and “F240500007”, respectively.

The data analysis was conducted based on the method used by Bruno et al. [11]. The “predict” function in R applies a fitted model to new data and returns predictions based on the estimated parameters. Once the model is trained, predictions for new observations are generated by applying the model equation, typically computed as the inner product of the model coefficients and the predictor values in the new data. Therefore, we refer to the output, derived using a modified Bruno method, as “AI vascular age”. The analysis followed the steps described below.

### 2.1. Deriviation of AI Vascular Age for Cohort I and Cohort II

#### 2.1.1. Explanatory Variable Selection, Multicollinearity Assessment, Spline Smoothing, and Deriving AI Vascular Age Using the Predict Function for Cohort I

**Figure 1:**
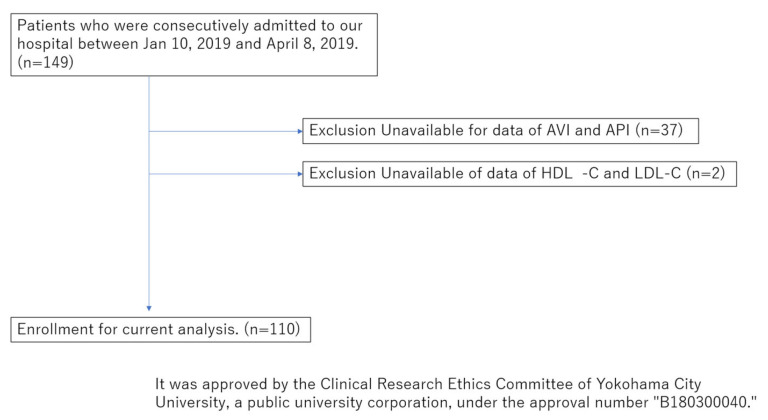
Flowchart showing inclusion and exclusion processes of the current study for Cohort I.

We installed the necessary statistical packages for R required for this study. From Cohort I (n = 149), subjects with missing data were excluded, and the final analysis was conducted on n = 110 (Figure 1, Table 1). As this was not part of the original data collection protocol, a number of cases were excluded from the analysis.

First, a step-wise regression analysis was performed with **Gender**, **BMI**, **HT**, **DM**, **HL**, **CKD**, **CAD**, **SBP**, **DBP**, **LDL-C**, **HDL-C**, **AVI**, **API**, **PR**, and **PP** as explanatory variables and **Chronological Age** as the dependent variable. After conducting stepwise selection, the **variance inflation factor (VIF)** was calculated to assess multicollinearity. A strong positive correlation was found between **SBP and API**. Given the measurement method of API, the observed positive correlation with SBP is not contradictory. It was determined that multicollinearity exists between SBP and API; therefore, one of them needed to be removed from the model. Since this study aimed to derive **AI Vascular Age** from pulse wave indices, **API was retained** in the model.

**Figure 2:**
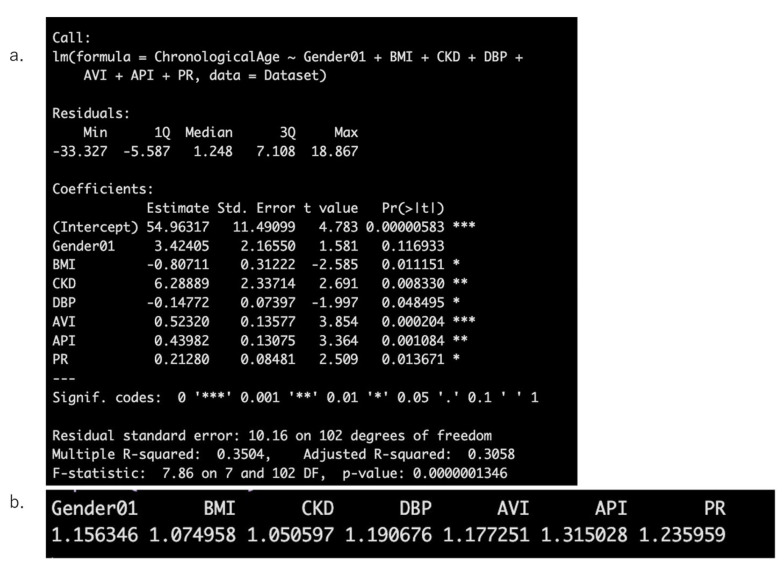
Results of stepwise regression analysis with chronological age as the dependent variable (**a**) and the calculation results of the Variance Inflation Factor (VIF) to assess multicollinearity (**b**).

Next, stepwise selection was performed again with **Gender**, **BMI**, **HT**, **DM**, **HL**, **CKD**, **CAD**, **DBP**, **LDL-C**, **HDL-C**, **AVI**, **API**, **PR**, and **PP** as explanatory variables and **Chronological Age** as the dependent variable after removing SBP. As a result, **Gender**, **BMI**, **CKD**, **DBP**, **AVI**, **API**, and **PR** were selected as explanatory variables. It was confirmed that none of these variables had a **VIF** > **2** (Figure 2a,b).

**Figure 3:**
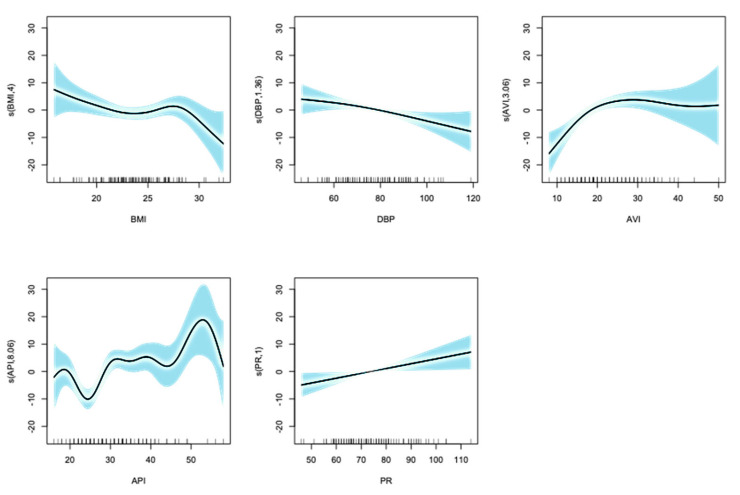
Results of the selected explanatory variables transformed using spline smoothing. Horizontal lines show each transformed variables and vertical lines show chronological age. The shaded area represents the 95% confidence interval. Whether the value of the smoothing function is positive or negative indicates the direction of the effect of the variable on the dependent variable (chronological age).

Then, the selected explanatory variables were transformed using **spline smoothing** following the method of Bruno et al. [11]. (Figure 3) and were then input into the “predict” function in R to derive AI vascular age.

#### 2.1.2. Derivation of AI Vascular Age for Cohort II

**Figure 4:**
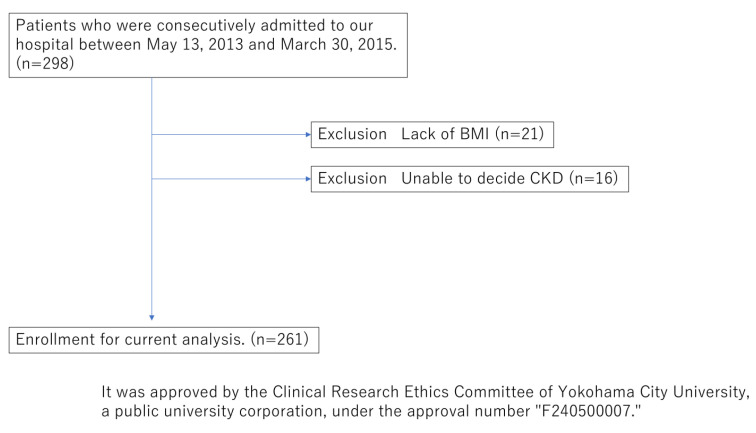
Flowchart showing inclusion and exclusion processes of the current study for Cohort II.

Subsequently, from May 2013 to March 2015, 261 out of the 299 outpatients in the Department of Cardiovascular Medicine at Yokohama City University Hospital, who had no missing data in the variables used, were included in the following study (Figure 4, Table 2). AI vascular age was successfully introduced using the vascular age prediction model constructed in the first cohort. We effectively derived AI vascular age in relation to chronological age in Cohort II, the same as in Cohort I.

Next, the relationship between the derived AI vascular age and chronological age was analyzed. The averages were compared, and the changes in age before and after derivation were visualized. The difference (ΔAge) between chronological age and AI vascular age, as well as the correlation with chronological age, were visualized using a scatter plot, and the regression equation was derived.

Then, to evaluate the performance of the derived AI-determined vascular age, comparisons were made between the top 20% and bottom 20% groups for cardiac diastolic function parameters measured using echocardiography (LV-DT, E/E’, E/A, E’), renal vascular sclerosis index (RI;Resistive Index), BNP, and eGFR using the Mann–Whitney U test (significance level *p* < 0.05). Simultaneously, the same evaluation items were compared between the top 20% and bottom 20% groups based on chronological age.

Statistical analysis was performed using R. The initial analysis was conducted on the first cohort, and the same variables were applied for a similar analysis in the second cohort.

## 3. Results

**Figure 5:**
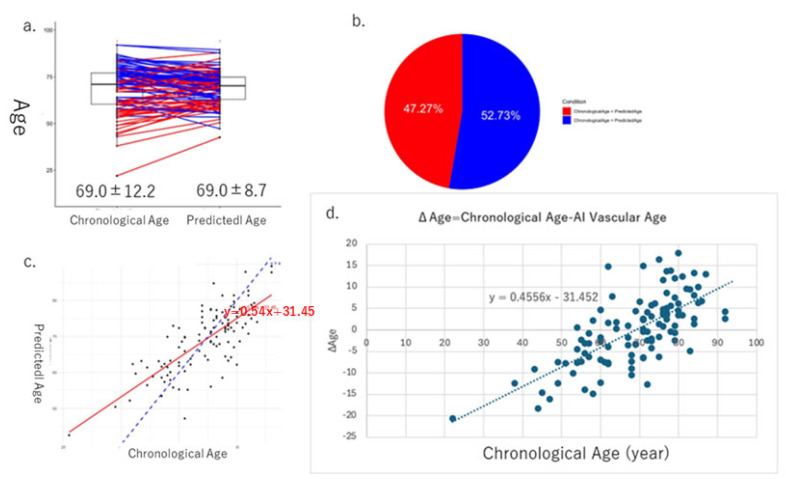
These figures show the results of AI vascular age in Cohort I. The average and standard error of chronological age is 69.0 ± 12.2, and that of AI vascular age is 69.0 ± 8.7, respectively. (**a**) In 52.7% of the cases, chronological age was higher than AI vascular age, whereas in 42.3% of the cases, AI vascular age was higher than chronological age (**b**). (**c**) Linear regression relationships between chronological age and AI vascular age. (**d**) Finally, there is a significant relationship between ΔAge (chronological age − AI vascular age) and chronological age.

The mean values of the derived AI vascular age (69.0 ± 8.7) and chronological age (69.0 ± 12.2) were calculated and compared in Cohort I, with no significant difference observed between them (Figure 5a). In 52.7% of the cases, chronological age was higher than AI vascular age, whereas in 42.3% of the cases, AI vascular age was higher than chronological age (Figure 5b). A positive correlation was observed between chronological age and AI vascular age (Figure 5c). A scatter plot of the difference between chronological age and AI vascular age (ΔAge) versus chronological age was created, and correlation analysis revealed a significant positive correlation (y = 0.4556x − 31.452, *p* < 0.05).

**Figure 6:**
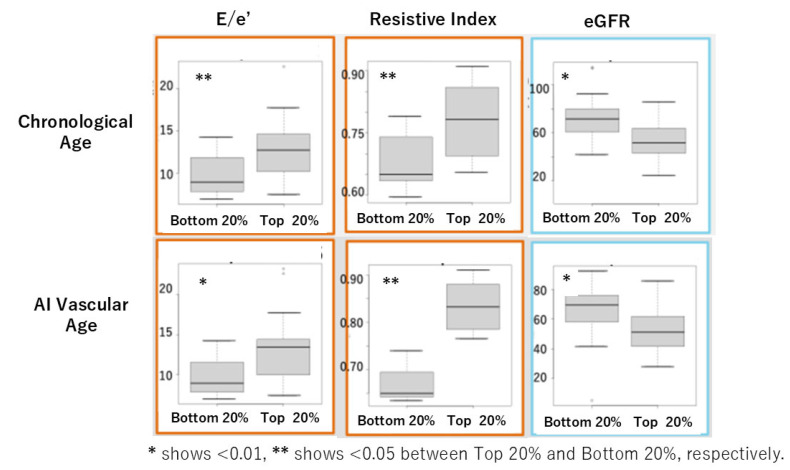
Comparison of cardiovascular markers and eGFR between the oldest 20% (**top**) and the youngest 20% (**bottom**) for chronological age and AI vascular age in Cohort I using the U-test.

In Cohort I, we evaluated the performance of chronological age and AI vascular age by comparing cardiovascular indicators and eGFR using heart and kidney ultrasound examinations. The comparison was made between the oldest 20% and the youngest 20% for each age group using the U-test. Significant differences due to aging were observed in E/e’, a marker of diastolic function, the RI (Resistive Index), a marker of renal artery resistance, and eGFR (Figure 6).

**Figure 7:**
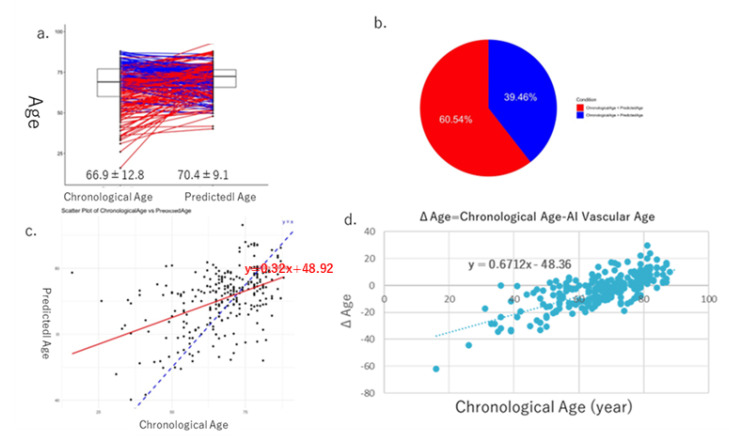
Results of AI vascular age in Cohort II. The average and standard error of chronological age is 66.9 ± 12.8, and that of AI vascular age is 70.4 ± 9.1. (**a**) In 52.7% of the cases, chronological age was higher than AI vascular age, whereas in 42.3% of the cases, AI vascular age was higher than chronological age (**b**). (**c**) Linear regression relationships between chronological age and AI vascular age. Finally, there is a significant relationship between ΔAge (chronological age − AI vascular age) and chronological age (**d**).

**Figure 8:**
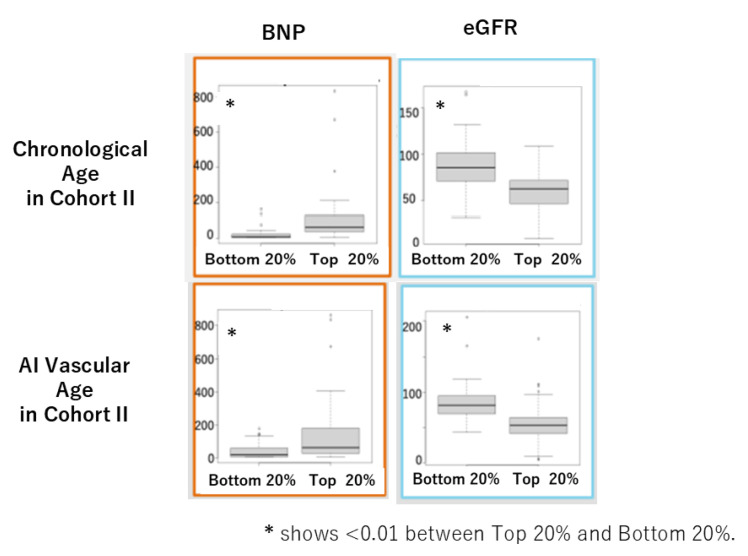
Comparison of BNP and eGFR between the oldest 20% (**top**) and the youngest 20% (**bottom**) for chronological age and AI vascular age in Cohort II using the U-test.

In Cohort II, the mean values of AI vascular age (70.4 ± 9.1) and chronological age (66.9 ± 12.8) were equivalent, as in Cohort I (Figure 7a,b), and a positive correlation was observed between the two (Figure 7c). Furthermore, a significant positive correlation was found between the difference ΔAge and chronological age (Figure 7d). When comparing cardiovascular markers and eGFR between the oldest 20% and the youngest 20% based on each age, using the U-test, we observed significant differences due to aging in BNP and eGFR (Figure 8).

The data in Table 1 are presented as means ± standard deviation or n (%), where: HT, hypertension; DL, dyslipidemia; DM, diabetes mellitus; IHD, ischemic heart disease; VHD, valvular heart disease; OMI, old myocardial infarction; CHF, chronic heart failure; RAS, renin–angiotensin system; BMI, body mass index; SBP, systolic blood pressure, DBP diastolic blood pressure; PR, pulse rate; AVI, arterial velocity pulse index; API, arterial pressure volume index; LDL-C, LDL cholesterol; HDL-C, HDL cholesterol; HbA1c, hemoglobin A1c; eGFR, estimated glomerular filtration rate; BNP, brain natriuretic peptide.

## 4. Discussion

In 2020, Bruno et al. attempted to derive AI vascular age using cf-PWV measurements and multiple variables based on two historical cohort datasets [11]. They investigated the prognostic implications of AI vascular age in these cohorts and identified three groups: (1) early vascular aging (EVA), characterized by advanced vascular aging, (2) normal vascular aging (NVA), representing an average aging pattern, and (3) supernormal vascular aging (SUPRENOVA), where vascular age was younger than chronological age and associated with a lower incidence of cardiovascular events. Their study demonstrated that AI vascular age performed consistently across the two independent cohorts. The “predict” function in R applies a fitted model to new data and returns predictions based on the estimated parameters. Once the model is trained, predictions for new observations are generated by applying the model equation, typically computed as the inner product of the model coefficients and the predictor values in the new data. Therefore, we refer to the output, derived using a modified Bruno method, as “AI vascular age,” as stated before in the Section 2.

In this study, we successfully derived AI vascular age using a modified version of Bruno et al.’s methodology [11] in two Japanese cohorts. Despite differences between the two cohorts, AI vascular age was derived based on multivariable models incorporating vascular indices without significant deviation from the population’s chronological age distribution. The difference between chronological age and AI vascular age (ΔAge) showed a significant linear correlation in both cohorts (Figure 5d and Figure 7d). This suggests that in chronic high-risk populations, such as patients attending cardiovascular outpatient clinics with a history of cardiovascular complications, younger individuals tend to exhibit accelerated vascular aging, whereas older individuals do not necessarily show advanced vascular aging. Furthermore, comparisons between high and low AI vascular age groups, as well as high and low chronological age groups, revealed significant differences in cardiac ultrasound indices, renal ultrasound indices, and estimated glomerular filtration rate (eGFR) (Figure 6 and Figure 8).

Since AI vascular age is derived from statistical models integrating vascular indices and representative cardiovascular disorder-related variables, it is potentially modifiable through medical interventions, distinguishing it from irreversible chronological age. Bruno et al. suggested that while chronological age cannot be medicalized, AI vascular age presents an opportunity for clinical application [11]. Our study demonstrated that AI vascular age can be derived in Japanese cohorts using AVI and API, following the methodology proposed by Bruno et al. [11]. The derived AI vascular age exhibited performance comparable to chronological age while offering a means to quantify residual risk associated with aging in a medically actionable manner.

Currently, the medical approach to atherosclerosis is largely focused on acute-phase care for catastrophic plaque rupture leading to thromboembolic events, such as acute coronary syndrome and ischemic stroke. Acute-phase treatments, including catheter interventions, intensive care, and surgical procedures, require substantial medical resources and incur significant healthcare costs. Furthermore, disparities in healthcare services exist between urban and rural areas due to differences in transportation infrastructure and available medical resources, despite Japan’s universal health insurance system.

Hypertension, diabetes mellitus, dyslipidemia, and smoking are established risk factors for atherosclerotic diseases. The efficacy of antihypertensive, antidiabetic, and lipid-lowering medications is evaluated not only by their ability to control blood pressure, glucose levels, and lipid profiles but also by their effectiveness in reducing cardiovascular complications, as demonstrated in Cardiovascular Outcome Trials (CVOTs). However, known and established risk factors account for only approximately 30% of cardiovascular complications [17]. Consequently, even with optimal risk factor management, around 70% of cardiovascular risk remains unaddressed. Identifying and appropriately targeting these residual risks represents an unmet medical need.

At present, atherosclerosis and arteriosclerosis are primarily medicalized when they manifest as an acute catastrophic event. Secondary prevention strategies, aimed at reducing recurrence in individuals who survive the acute phase, typically involve lifelong pharmacological interventions, such as antiplatelet and anticoagulant therapies, in addition to conventional risk factor management. However, these approaches do not provide a curative solution [18]. Enhancing the precision of primary prevention for atherosclerosis could enable a shift from a high-risk strategy to a population-wide risk reduction strategy.

Hypertension, which affects approximately 40 million people in Japan, serves as a model for a successful population-wide approach. With the development of various antihypertensive drugs, the management of hypertension has reached a mature phase. By providing effective antihypertensive medications, Japan has successfully reduced the incidence of hemorrhagic stroke through a highly inclusive population-based strategy.

The key challenges in medicalizing atherosclerosis and arteriosclerosis lie in overcoming the irreversible risk posed by aging and addressing the escalating social, human, and economic costs associated with an acute-phase healthcare system in a rapidly aging society [5,19]. If atherosclerosis and arteriosclerosis can be incorporated into a population-wide prevention framework similar to hypertension, it may be possible to significantly reduce the incidence of atherosclerotic cardiovascular complications.

Various stiffness indices and vascular endothelial function indicators have been developed to medicalize pre-clinical atherosclerosis [5,7,20]. Among these, pulse wave indices are considered a modality capable of visualizing pre-clinical and latent atherosclerosis and arteriosclerosis. Existing pulse wave indices can be broadly classified into arterial stiffness indices and vascular endothelial function indices. In Japan, a wide variety of pulse wave indices have been developed, leading to a situation where multiple methods coexist without standardization. These indices have suffered from technical limitations, variations in measurement methodologies, and a lack of reproducibility, preventing their standardization and clinical implementation, ultimately leading to their obsolescence.

In contrast, in Western countries, carotid–femoral pulse wave velocity (cf-PWV) is considered the standard stiffness index [5,21]. cf-PWV has several advantages as a vascular marker: (1) it is a simple and minimally invasive measurement, (2) it is cost-effective, (3) it correlates with pathological conditions, (4) it exhibits high accuracy and reproducibility, (5) it allows for standardized measurement methods, and (6) it has prognostic predictive capability. However, its widespread application is hindered by the requirement for skilled operators, the necessity for the subject to be in a supine position, and the limited availability of measurement in clinical settings. Additionally, a major limitation is the lack of specific medical interventions that directly target cf-PWV, making it uncertain whether improvements in cf-PWV necessarily lead to better prognostic outcomes [22,23].

Future research should focus on investigating the relationship between AI vascular age and clinical outcomes across multiple cohorts. Additionally, efforts should be made to standardize the derivation and application of AI vascular age in the Japanese population to facilitate its clinical implementation. As we discussed above, in Japan, a wide variety of pulse wave indices have been developed, leading to a situation where multiple methods coexist without standardization. Introducing AI vascular age into clinical usage, with indices including AVI and API, might become a commodity when medicalizing anti-aging medicine in the future.

## 5. Conclusions

This study introduces a novel approach to estimating AI-determined vascular age using cuff-based indices—arterial velocity pulse index (AVI) and arterial pressure volume index (API)—instead of traditional cf-PWV, within a Japanese hospital-based population.

Our findings demonstrated that AI vascular age correlates significantly with chronological age and may reflect subclinical cardiovascular and renal aging. Notably, individuals classified as having higher AI vascular age exhibited significant differences in diastolic function (E/e’), renal vascular resistance (RI), and renal function (eGFR) compared to those with lower vascular age. These results were consistent across two independent cohorts and suggest the potential of this method for early risk stratification in clinical practice.

However, several limitations should be acknowledged. First, the study design was retrospective and exploratory in nature, based on a single-center dataset with a relatively modest sample size. Second, the AI model was trained and applied within a specific population, which may limit generalizability to other ethnic or clinical backgrounds.

Future research should involve prospective, multicenter studies with larger and more diverse populations to validate the utility and predictive power of AI vascular age based on AVI and API. In addition, longitudinal studies are needed to determine its role in predicting long-term cardiovascular outcomes. Integration with other biomarkers and machine learning techniques may further refine vascular aging assessment and support personalized preventive strategies.

## Figures and Tables

**Table 1:** Summary of general clinical characteristics of Cohort I.

	Overall	Female	Male
n	110	40	70
Age (Chronological)	69.0 ± 12.2	68.9 ± 13.0	69.1 ± 11.8
BMI	23.7 ± 3.2	22.9 ± 3.2	24.2 ± 3.2
Hypertension (%)	66 (60.0%)	25 (62.5%)	41 (58.6%)
DM (%)	30 (27.3%)	12 (30.0%)	41 (58.6%)
HL (%)	31 (28.2%)	13 (32.5%)	18 (25.7%)
CKD (%)	26 (23.6%)	6 (15.0%)	20 (28.6%)
IHD (%)	32 (29.1%)	11 (27.5%)	21 (30.0%)
SBP (mmHg)	135.9 ± 22.6	136.7 ± 19.8	135.4 ± 24.2
DBP (mmHg)	77.6 ± 14.4	75.3 ± 14.8	79.0 ± 14.0
PR (bpm)	73.9 ± 12.8	74.4 ± 12.7	73.6 ± 12.9
AVI	22.3 ± 7.8	23.0 ± 8.3	21.9 ± 7.5
API	30.9 ± 8.5	33.5 ± 8.1	29.4 ± 8.5
eGFR (mL/min/1.73^2^)	60.9 ± 16.3	63.5 ± 15.4	59.3 ± 16.7
HbA1c (%)	6.1 ± 0.7	6.2 ± 0.8	6.0 ± 0.5
BNP (pg/mL)	72.0 ± 154.9	78.8 ± 212.5	67.8 ± 108.5
LDL-C (mg/dL)	106.6 ± 33.2	112.8 ± 39.5	103.0 ± 28.8
HDL-C (mg/dL)	63.7 ± 20.4	70.5 ± 24.5	59.7 ± 16.6

**Table 2:** Baseline characteristics of Cohort II.

	Overall	Female	Male
C	261	94	167
Age (Chronological)	66.9 ± 12.8	66.1 ± 14.1	67.4 ± 12.0
BMI	23.1 ± 3.6	22.7 ± 3.9	23.3 ± 3.4
Hypertension (%)	159 (60.9%)	57 (60.6%)	102 (61.1%)
DM (%)	55 (21.1%)	12 (37.2%)	43 (25.7%)
HL (%)	116 (44.4%)	35 (37.2%)	81 (48.5%)
CKD (%)	72 (27.6%)	22 (23.4%)	50 (29.9%)
SBP (mmHg)	132.5 ± 21.5	134.1 ± 24.0	131.2 ± 19.9
DBP (mmHg)	73.7 ± 13.6	71.9 ± 14.7	74.8 ± 12.8
PR (bpm)	75.0 ± 13.0	77.6 ± 14.6	74.8 ± 12.8
AVI	23.5 ± 8.2	24.9 ± 8.7	22.7 ± 7.9
API	30.8 ± 7.8	33.2 ± 8.0	29.5 ± 7.3
eGFR (mL/min/1.73^2^)	60.9 ± 16.3	76.4 ± 29.0	69.1 ± 23.4
HbA1c (%)	6.1 ± 1.0	6.2 ± 1.1	6.1 ± 0.9
BNP (pg/mL)	71.7 ± 25.9	47.2 ± 48.7	103.2 ± 164.6
LDL-C (mg/ dL)	108.1 ± 35.5	114.8 ± 34,7	104.6 ± 35.5
HDL-C (mg/dL)	61.5 ± 19.1	65.7 ± 21.4	59.6 ± 17.4

Data are presented as mean ± standard deviation or n (%).DM, diabetes mellitus; BMI, body mass index; SBP, systolic blood pressure, DBP diastolic blood pressure; PR, pulse rate; AVI, arterial velocity pulse index; API, arterial pressure volume index; LDL-C, LDL cholesterol; HDL-C, HDL cholesterol; HbA1c, hemoglobin A1c; eGFR, estimated glomerular filtration rate; BNP, brain natriuretic peptide.

## Data Availability

The original contributions presented in the study are included in the article, further inquiries can be directed to the corresponding authors.

## Abbreviations

The following abbreviations are used in this manuscript:

AVI	Arterial velocity pulse index
API	Arterial pressure volume index
PWV	Pulse wave velocity
CKD	Chronic kidney disease
